# A pressing need to respond to the needs and sexual and reproductive health problems of adolescent girls living with HIV in low- and middle-income countries

**DOI:** 10.7448/IAS.18.6.20297

**Published:** 2015-12-01

**Authors:** Venkatraman Chandra-Mouli, Alice Armstrong, Avni Amin, Jane Ferguson

**Affiliations:** 1 Department of Reproductive Health and Research, World Health Organization, Geneva, Switzerland; 2 Department of HIV/AIDS, World Health Organization, Geneva, Switzerland; 3 Department of Maternal, Newborn, Child and Adolescent Health, World Health Organization, Geneva, Switzerland

**Keywords:** adolescents living with HIV, adolescent girls, sexual and reproductive health

## Abstract

**Introduction:**

This commentary provides the rationale and makes a call for greater investment and effort to meet the sexual and reproductive health (SRH) problems of adolescent girls living with HIV in low- and middle-income countries (LMIC).

**Discussion:**

Adolescent girls in LMIC are at a greater risk of acquiring HIV infection than their male peers. They also face a number of other serious SRH problems – early pregnancy, pregnancy- and childbirth-related complications, unsafe abortions, sexual abuse and intimate partner violence and sexually transmitted infections. While many LMIC have made notable progress in preventing HIV in children and adults and in improving the access of these population groups to HIV treatment and care, adolescents in general and adolescent girls in particular have not received the same effort and investment.

**Conclusions:**

Much more needs to be done to implement proven approaches to prevent new HIV infections in adolescent girls in LMIC and to meet the needs of those living with HIV.

## Introduction

In the 15 years since the United Nations Millennium Declaration, the world has made tremendous progress in reducing the number of new HIV infections and in reducing deaths in those living with HIV [1,2].

Relative to this improvement, there has been limited progress in preventing new HIV infections in adolescents, especially in low- and middle-income countries (LMIC) [1,2]. This is both because the interventions that have been used have been shown to be of limited effectiveness [3] and because HIV prevention efforts in adolescents have not received the investment and effort needed [4]. Many LMIC have made substantial progress in diagnosing and treating children and adults with HIV infection. Lack of concerted efforts directed at adolescents has meant that LMIC have made relatively less progress in reaching this population group with HIV testing and counselling, as well as treatment and care services [1,5,6].

While HIV infections and HIV-related mortality and morbidity occur in both adolescent boys and girls, the number of new infections and the number of individuals living with HIV are far higher in the latter [1,2,5,6]. This is primarily because biological as well as social, cultural and economic factors increase girls’ vulnerability to HIV infection and to its health and social consequences. While acknowledging that boys and young men need concerted attention too, the focus of this paper is on adolescent girls and young women, with a special focus on those living with HIV. It provides the rationale and makes a call for greater investment and effort to meet the sexual and reproductive health (SRH) problems of adolescent girls living with HIV in LMIC.

## Discussion

This paper begins by describing the SRH problems that adolescent girls face. It then discusses the limited progress that has been made in preventing these problems, responding to them when they occur and especially in addressing the social, cultural and economic drivers of these problems. It then discusses the need to increase access to HIV testing and counselling for adolescent girls and to treatment and care for those living with HIV.

## SRH problems in adolescent girls

While most adolescents make the transition to adulthood in good health, not all are so fortunate. They face SRH problems; in endemic countries, HIV is an important one of them.

Globally, new HIV infections in all age groups declined by 38% since 2001 [6]. New HIV infections among adolescents declined even more sharply, by 43%. However, a substantial number of HIV infections – an estimated 250,000 new infections – occurred in adolescents globally in 2012 (range 210,000 to 290,000). Progress was uneven across different regions. For example, while new infections have decreased substantially in Eastern and Southern Africa, they have remained stable in Asia and the Pacific [1,5,6]. Adolescent girls are affected more than their male peers; 64% of the new infections were in girls aged 15 to 19 [1,5,6]. Finally, data from the few available studies on adolescent girls in key populations (Note: Key populations (also referred to as *most-at-risk populations*) are people who inject drugs, gay men and other men who have sex with men, transgender persons and sex workers. They are disproportionately infected with HIV compared to the general population.) suggest that they have significantly higher HIV prevalence and other sexually transmitted infections (STIs) than their peers in the general population [7,8].

Adolescent girls are affected by a number of other SRH problems. An estimated 16 million girls aged 15 to 19 and some one million girls less than 15 years give birth every year – most in LMIC [9,10]. Complications during pregnancy and childbirth are the second leading cause of death for 15 to 19 year-old girls globally (after road traffic injuries) [9]. Adolescent pregnancy, especially when it occurs in early adolescence, is associated with higher rates of maternal mortality and morbidity [11]. It is also associated with increased risks of mortality and morbidity in newborns and children [12]. Of the estimated 22 million unsafe abortions that occur every year, 15% occur among girls aged 15 to 19 [13]. Adolescent girls are at risk of childhood sexual abuse in homes and community settings such as schools, from influential adults around them. Globally, 1 in 10 girls under the age of 20 have been sexually abused [14]. Additionally, an estimated 30% of girls aged 15 to 19 experience intimate partner violence [15]. The WHO estimates that every year 500 million people become ill with one of four curable STIs – chlamydia, gonorrhoea, syphilis and trichomoniasis. In addition there is a huge burden of viral STIs. The WHO also estimates that more than 530 million people are infected with the herpes simplex virus, which causes genital herpes, and more than 290 million women with the human papilloma virus, which causes genital warts and cervical cancer. The available data is not disaggregated by age and so we do not have global estimates for STIs in adolescents. However, in most populations higher levels of infection are noted in young populations [16].

Globally, there are an estimated 35 million people living with HIV. In 2013, an estimated 1.5 million died of AIDS-related illnesses. This figure represents a 35% decline since the peak of 2.4 million deaths, recorded in 2005. AIDS-related deaths, however, have not decreased among adolescents aged 10 to 19 [2]. At the end of 2013, an estimated 2.1 million adolescents (aged 10 to 19 years) were living with HIV; with the majority in sub-Saharan Africa [6]. This estimate includes both perinatally infected children surviving into adolescence as well as individuals infected in the second decade of their lives. In 2013, an estimated 120,000 adolescents aged 10 to 19 years (range 100,000 to 130,000) died of AIDS-related illnesses [1,5]. AIDS remains the number one killer of adolescents in sub-Saharan Africa [2].

## Meeting the SRH information and service needs of all adolescent girls and of those living with HIV

In many contexts, adolescent girls are ill-informed about their bodies and their health and are unprepared for the changes they are experiencing and the challenges they could face. For example, only 30% of young women aged 15 to 24 in sub-Saharan Africa countries (with available data) had comprehensive, correct knowledge of HIV transmission in 2014. This represents an increase of less than 10% in four years [2]. Improving adolescent girls’ knowledge and understanding of SRH and building their life skills to take charge of their health is a crucial step in meeting their health needs and fulfilling their rights [17]. As girls move from older childhood into and through adolescence and then into early adulthood, they need sexuality education that responds to their developmental stages and circumstances and evolves with their changing needs. The sexuality education they receive should be comprehensive; it should both include and go beyond information on sexuality, reproduction and SRH problems and how to avoid them [18,19].

Adolescent girls living with HIV must learn what all girls need to. In addition they must learn to live with a chronic illness and to deal with the emotional and psychological issues associated with the knowledge that HIV is a highly stigmatized transmissible infection. This aspect often has an enormous impact on their sexual health and relationships. Adolescent girls living with HIV do not lose their desire for sex or to have families. Some have not yet had sex but hope/intend to do so in the future and have questions and concerns. Many others have had sex and have unanswered questions and concerns about infecting others, disclosing their status to their partners and having sex and children safely. They need information and counselling that responds to these needs [20,21].

Almost universally, adolescent girls lack access to the SRH commodities (such as condoms and other contraceptives) that they need to protect themselves or to the health services they need (e.g. diagnosis and treatment of STIs, HIV testing and counselling, treatment and care). Restrictive laws and policies in many countries forbid the provision of these services to unmarried adolescents, those below the age of majority. Often adolescent girls do not know where and how to obtain the health services they need. Even when they are able to obtain health services, they are often reluctant to do so because of fears about privacy, confidentiality, of being judged and of being treated with disrespect [17]. And as a study by Nalwadda *et al*. in Uganda highlighted, when they do seek care, adolescent girls say that they are treated with disrespect or are even turned away by providers who refuse to offer them services [22].

Adolescents living with HIV face an additional burden. Stigma and discrimination against people living with HIV and key populations in healthcare settings continue to undermine HIV responses [23]. Adolescent girls can be labelled as promiscuous or discriminated against by not being provided the health services they need [23]. Further, in some settings, they can be coerced into procedures such as sterilization or pregnancy termination [24].

Whether or not they are living with HIV, adolescent girls have a right to a healthy, enjoyable and safe sex life. SRH services should enable them to reduce or avoid health problems and should enable those who experience health problems to recover [25,26]. Countries should eliminate medical and social restrictions to the provision of SRH services including contraceptives to adolescent girls and should support and enable them to obtain commodities and services that are appropriate to their needs and preferences through delivery mechanisms that are acceptable to them [27]. Health services should also be ready to provide those girls who are pregnant with quality antenatal care including linkages to prevention of mother-to-child transmission of HIV care or to help in obtaining a safe abortion where this is permitted by law. Effective care during childbearing is important to ensuring the survival of mothers and their babies and the prevention of mother-to-child transmission and other problems such as fistulas resulting from obstructed labour. Adolescent girls living with HIV need access to contraceptive services including emergency contraception, safe abortion services and services to prevent mother-to-child transmission of HIV, if they get pregnant and want to continue with their pregnancies. They also need information and services to avoid STIs and diagnosis and treatment for STIs when they occur.

## Addressing the social, cultural and economic drivers of SRH problems in all adolescent girls and in those living with HIV

In several settings, adolescent girls engage in sexual relationships with men who are several years older than them. These relationships entail the exchange of sex for essentials (such as food and school fees) as well as non-essential ones (such as mobile phones). Cross-generational sexual relationships are a key driver of HIV risk among adolescent girls. To respond to this, a number of research studies and projects have been undertaken to test approaches to reduce the vulnerabilities of adolescent girls through economic empowerment interventions such as waivers of school fees, cash transfers and microfinance schemes. There is a growing knowledge base in this area [28].

There is also growing research evidence and project experience in preventing sexual coercion of girls [29,30]. This endeavour requires a multifaceted and multilevel approach. At the wider societal level, laws that forbid violence against women and girls should be passed and enforced effectively. Together with this action, efforts are needed to develop social norms that are intolerant of such violence. At the community level, steps should be taken to protect girls and women from physical and emotional violence at home, as well as sexual harassment and coercion in educational institutions and other community settings. These efforts should include activities directed at boys and men to address masculine norms that encourage boys and young men to take sexual risks to prove their manhood and to assert their masculinity by coercing girls and women to have sex. Adolescent girls and boys should be taught that violence is an unacceptable way to resolve conflicts. Moreover, boys and young men should be taught that sexual relationships should be based on equity and respect [29,30].

Studies from many countries point to the common occurrence of forced sex involving adolescent girls and the HIV risk associated with it [31]. There is a dearth of information on whether adolescent girls with HIV experience coerced sex or pressure to have transactional sex. However, such a situation is not unlikely. Given this information, adolescent girls living with HIV should be included in empowering interventions directed at girls uninfected with HIV.

## Increasing access to HIV testing and counselling for adolescent girls and to treatment and care for those living with HIV

As indicated earlier, there are an estimated 1.2 million adolescents living with HIV, with 70 to 80% living in sub-Saharan African. Many of the latter are unaware of their HIV status [2,5]. While there are currently no estimates of antiretroviral coverage for adolescents, the available evidence suggests higher rates of loss to follow-up, poor adherence and increased requirement for psychosocial support in this age group [32–36]. Studies have also pointed to gaps and weaknesses in health services to address the SRH needs of adolescents living with HIV [37–39]. For example, a Kenyan study of adolescents living with HIV reported low rates of contraceptive use (66%). Of those sexually active female respondents, 68% had already been pregnant and three-quarters of those were unintended pregnancies [38]. Additionally, higher mother-to-child transmission risk was seen among infants of HIV-positive adolescent mothers compared to adult mothers, with a 1.7-fold increased risk compared to adult mothers in one study in KwaZulu Natal, South Africa [39]. Adolescent girls from key population groups are also often subject to significant levels of stigma, discrimination, violence, increased risk for criminalization and incarceration and social isolation. These further exclude them from essential platforms for support and access to SRH and other prevention or treatment services [23].

## Looking back at the global response to meeting the needs and fulfilling the rights of adolescent girls, 21 years since the International Conference on Population and Development, and 15 years since the United Nations Millennium Declaration

In 2014, the world commemorated the twentieth anniversary of the International Conference on Population and Development (ICPD). Reviews of progress in the implementation of adolescent sexual and reproductive health policies and programmes over the two decades since the landmark conference noted that progress was limited and patchy [40]. The Millennium Development Goals report for 2015 also points to limited progress made in adolescents in relation to Goals 5 and 6 [2].

A review of the ICPD's achievements that focused on adolescent girls echoed these findings: “… many countries have yet to make significant progress in delaying marriage and childbearing, reducing unintended childbearing, narrowing gender disparities that put girls at risk of poor sexual and reproductive health outcomes, expanding health awareness or enabling access to sexual and reproductive health services” [41].

Given this backdrop, UNAIDS has called for a much stronger collective response to preventing HIV and HIV-related mortality in adolescents [42].

Two recent reviews stress the need to strengthen HIV prevention efforts directed at adolescent girls in LMIC, by using tailor-made combinations of biomedical, behavioural and structural interventions to address the web of individual and environmental factors that increase their vulnerability to HIV. Both stress the importance of developing more effective prevention options [28,43].

HIV prevention efforts must be implemented with efforts to improve the provision of, access to and uptake of quality health services (including SRH services, HIV testing counselling and HIV treatment and care services) to adolescent girls living with HIV. Two complementary approaches are needed. Firstly, lack of the required competencies and moralistic attitudes or values stand in the way of health workers providing adolescents living with HIV with the health services they need. Training, value clarification and ongoing support can enable them to make the contributions they need to. Two important starting points are making an assessment of sexual activity and providing full and accurate information [44]. Secondly, in most LMIC, health services are either not available or are not geared towards adolescents. Existing services should be made more welcoming (accessible and acceptable) to adolescents living with HIV – including those in key populations. Integrating SRH services with other health services, decentralizing their provision away from hospitals to clinics in the adolescent's neighbourhood and reaching out with services in the community can extend their range [45].

## Conclusions

Limited progress has been made in preventing SRH problems, notably HIV infections, in adolescent girls and in preventing HIV-related mortality in this group. This inadequate improvement, especially in relation to the progress made in other groups, is unacceptable. Much more needs to be done to prevent HIV in girls and to enable those living with HIV to survive and live to their full potential.

## Author information

Venkatraman Chandra-Mouli works as a scientist on adolescent sexual and reproductive health in the WHO's Department of Reproductive Health and Research, which includes the Special Programme of Research, Development and Research Training in Human Reproduction. His work includes supporting efforts to build the epidemiologic and evidence base and supporting countries to translate this evidence into action. He has led or contributed substantially to a number of the WHO's publications on adolescent health, including evidence reviews, policy and programmatic guidance, advocacy documents, training and self-learning materials and evaluation tools. As part of his work in the WHO, he has contributed to the work of national-level bodies and front-line organizations, in many different social, cultural and economic settings.

Alice Armstrong is a nurse practitioner and prescriber working in HIV and sexual reproductive health with a particular focus on adolescents. She is currently an affiliated consultant in the HIV/AIDS Department in the WHO in Geneva. She has coordinated the development of the HIV Testing and Counselling online implementation tool and the young key populations’ technical briefs; she has also worked on the adolescent components of the consolidated key population and antiretroviral guidance. She is currently working on adolescent antiretroviral treatment and adolescent male circumcision. Before joining the WHO, she was the adolescent programme coordinator for the Children's HIV Association, South Africa, providing technical support to the Ministry of Health on adolescent HIV service delivery.

Jane Ferguson is a scientist in the Maternal, Newborn, Child and Adolescent Health Department of the WHO in Geneva. She is the focal point for adolescent health and responsible for research and development related to adolescents in that Department. Jane has held a variety of positions in various configurations of the WHO over the past several decades, devoted to moving adolescent health onto the public health agenda. During these years she has worked with ministries of health and civil society organizations, including youth organizations, United Nations partners and technical and funding agencies in countries in all regions of the world. She has led adolescent health strategy development, defined research priorities and developed programme support tools, including recent guidelines on adolescent reproductive health outcomes and HIV prevention, treatment and care.

Avni Amin is a technical officer for violence against women at the Department of Reproductive Health and Research, WHO Geneva. She has worked on issues of gender equality, violence against women and their linkages to sexual and reproductive health and HIV for the last 18 years.
